# PrankWeb 4: a modular web server for protein–ligand binding site prediction and downstream analysis

**DOI:** 10.1093/nar/gkaf421

**Published:** 2025-05-19

**Authors:** Lukáš Polák, Petr Škoda, Kamila Riedlová, Radoslav Krivák, Marian Novotný, David Hoksza

**Affiliations:** Department of Software Engineering, Faculty of Mathematics and Physics, Charles University, Prague, 121 16, Czech Republic; Department of Software Engineering, Faculty of Mathematics and Physics, Charles University, Prague, 121 16, Czech Republic; Department of Software Engineering, Faculty of Mathematics and Physics, Charles University, Prague, 121 16, Czech Republic; Department of Software Engineering, Faculty of Mathematics and Physics, Charles University, Prague, 121 16, Czech Republic; Institute of Organic Chemistry and Biochemistry of the Czech Academy of Sciences, Prague, 160 00, Czech Republic; Department of Cell Biology, Faculty of Science, Charles University, Prague, 128 00, Czech Republic; Department of Software Engineering, Faculty of Mathematics and Physics, Charles University, Prague, 121 16, Czech Republic

## Abstract

Knowledge of protein–ligand binding sites (LBSs) is crucial for advancing our understanding of biology and developing practical applications in fields such as medicine or biotechnology. PrankWeb is a web server that allows users to predict LBSs from a given three-dimensional structure. It provides access to P2Rank, a state-of-the-art machine learning tool for binding site prediction. Here, we present a new version of PrankWeb enabling the development of both client- and server-side modules acting as postprocessing tasks on the predicted pockets. Furthermore, each module can be associated with a visualization module that acts on the results provided by both client- and server-side modules. This newly developed system was utilized to implement the ability to dock user-provided molecules into the predicted pockets using AutoDock Vina (server-side module) and to interactively visualize the predicted poses (visualization module). In addition to introducing a modular architecture, we revamped PrankWeb’s interface to better support the modules and enhance user interaction between the 1D and 3D viewers. We introduced a new, faster P2Rank backend or user-friendly exports, including ChimeraX visualization.

## Graphical Abstract

**Graphical Abstract:**
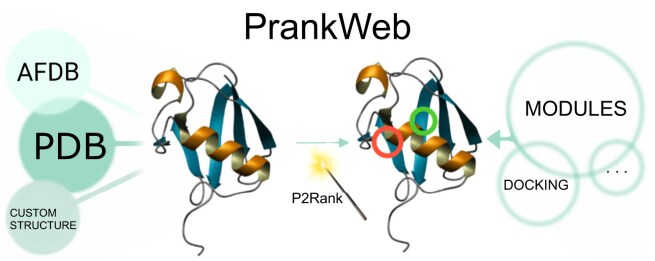

## Introduction

Proteins are central to virtually all biological processes, exerting their functions through interactions with other molecules. A particularly important class of these interactions involves small molecules, such as metabolites, signaling compounds, or drug candidates. Identifying the regions on proteins where small molecules bind—known as ligand binding sites—is crucial for understanding molecular mechanisms and enabling applications in fields like bioengineering and drug discovery [1–4].

Over the years, various computational methods have been developed to predict protein–ligand binding sites. Early approaches relied on geometric and physicochemical features [5, 6], and were later followed by machine-learning models such as P2Rank [7]. More recently, deep learning has gained momentum in this field [8–12], although many such tools predict binding residues rather than complete binding sites [13]. Interestingly, the top-performing traditional and machine-learning-based approaches still remain competitive, especially when benchmarked on independent datasets [13].

Despite this growing interest, benchmarking results from both independent studies and papers introducing deep learning methods show that the best-performing traditional methods, such as P2Rank, are still on par with—or even outperform—many recent deep learning approaches [12, 13] (we particularly recommend [13] for an independent comparison with the latest deep learning methods). While a detailed analysis of this phenomenon is beyond the scope of this paper, we believe that issues related to data curation—including potential data leakage and limited diversity of training data—may be contributing factors, particularly when considering performance on well-curated and independent benchmarks [13].

Among the machine-learning approaches, P2Rank stands out for its accuracy and usability. It forms the core of PrankWeb, a widely used web application that extends P2Rank predictions with evolutionary conservation and provides a user-friendly interface for binding site exploration. PrankWeb supports protein structures from the PDB, AlphaFold models retrieved via UniProt IDs, and user-uploaded structures, making it a versatile tool for researchers working on diverse proteins.

However, binding site detection is rarely the final goal. It is typically followed by additional postprocessing steps—collectively referred to as downstream or pocket postprocessing tasks—which encompass anything the user does with the prediction. While docking a molecule of interest into the predicted site is a common example, downstream tasks can vary. For instance, one might search for homologous protein–ligand complexes with overlapping binding sites [14] or identify tunnels originating from the predicted site [15].

These downstream applications motivated the extension of PrankWeb, which is presented here. We restructured PrankWeb to support modular postprocessing tasks on predicted pockets. These tasks can range from simple computations, such as pocket volume estimation, to more complex, time-intensive operations, such as docking user-provided molecules into predicted binding sites. Each module can be paired with a visualization component, providing an interactive and user-friendly exploration experience. The new system has been used to integrate docking capabilities via AutoDock Vina, allowing PrankWeb users to dock molecules into predicted pockets and visualize predicted poses interactively.

Beyond the modular architecture, PrankWeb’s interface has been redesigned to better support these extensions and improve integration between its 1D and 3D viewers. The current version also introduces a faster P2Rank backend, prediction versioning, AlphaFold model coloring based on pLDDT values, and enhanced data export options, including support for UCSF ChimeraX visualization [16].

Despite existing efforts to develop web servers for predicting general protein–ligand binding sites [17], docking molecules into predefined locations [18–20], or even a combination of these [21, 22], many of these tools are focused on one of the tasks, are unavailable, are no longer actively maintained, or lack a robust set of state-of-the-art prediction and analytical features. In contrast, PrankWeb stands out with its well-maintained infrastructure, user-friendly interface, and strong potential for future development—thanks to its newly updated, extensible framework.

## Pocket postprocessing modules

As described earlier, many PrankWeb users seek to further analyze pockets by applying various postprocessing steps, such as molecular docking. To enhance user experience, we have implemented functionality that allows these tasks to be performed directly within the web interface.

We have implemented interfaces for two types of tasks—client- and server-side tasks. Client-side tasks are executed directly in the user’s browser. An example of this is calculating pocket volume—when such a calculation is required, it is lightweight enough to be handled on the client side. In contrast, docking is computationally intensive and demands substantial resources. For such cases, we have implemented server-side tasks. These tasks leverage the existing P2Rank prediction architecture and utilize Celery job queues. Each server-side task is encapsulated within a dedicated Docker image, which is integrated into the architecture using Docker Compose.

The results of these postprocessing tasks can be either displayed directly to the user or downloaded as a ZIP file. To eliminate the need for external visualization tools, we have developed another interface that enables the development of modules supporting in-app visualization of task results. The visualization adopts a design consistent with the P2Rank predictions, ensuring a smooth user experience.

To avoid multiple executions of computationally expensive tasks, PrankWeb implements a caching mechanism that assigns a unique hash to each task. This hash is determined by the structure, pocket, and task-specific parameters. For docking, this includes the SMILES (simplified molecular input line entry system) representation of the docked molecule. Therefore, the docking of a particular molecule into a particular pocket of a given PDB structure is carried out only once, and the results are shared among users. Additionally, the task history for individual users is stored in the browser, allowing easy access to the previously computed results.

When deciding which modules to include in PrankWeb, we analyzed how predicted pockets are used by researchers based on citation patterns in papers referencing PrankWeb. We also distributed a questionnaire to gather direct user feedback on desired postprocessing features. Docking was consistently identified as the most common next step after binding site prediction and emerged as the most frequently requested addition. Based on this insight, we prioritized docking over other functionalities. We are currently developing a new module for detecting tunnels that originate from predicted pockets. Looking ahead, we are also considering adding functionality to analyze the flexibility of predicted pockets, for example, through general molecular dynamics simulations. At present, PrankWeb offers two modules: pocket volume computation (client-side) and docking (server-side).

While pocket volume computation can be performed relatively straightforwardly by considering the convex hull encompassing the pocket atoms, the docking process for a molecule of interest is more complex. The following section will discuss the docking pipeline in detail.

### Molecular docking

Molecular docking is performed using AutoDock Vina [23, 24], an efficient and widely used docking tool for predicting ligand–protein interactions. The docking workflow is containerized using Docker, ensuring reproducibility, portability, and ease of deployment across different computing environments. The module is structured as a GitHub repository (https://github.com/kiarka7/DODO) and encapsulated within a Docker container, allowing streamlined execution and seamless integration into the PrankWeb platform.

To further improve accuracy and usability, several modifications are implemented on top of the standard AutoDock Vina workflow. The docking parameters, including the receptor, ligand, box center, box size, and exhaustiveness, are managed through a structured JSON input file. The docking box parameters are automatically determined based on P2Rank pocket predictions. Specifically, P2Rank provides the pocket center, while the bounding box size is dynamically calculated by measuring the distance of the furthest surface atom from the pocket center and adding a 5 Å buffer to enhance accuracy.

Before the actual docking, both the receptor and ligand structures undergo preprocessing to ensure compatibility with AutoDock Vina. The receptor is first cleaned by removing non-essential molecules, redundant atoms, and alternative conformations. Hydrogen atoms are then added using MGLTools [25] via the prepare_receptor4.py script, which optimizes hydrogen bonding interactions. The ligand input is required in SMILES format, which is converted to a 3D structure in PDB format using OpenBabel [26]. Subsequently, the ligand is prepared using the prepare_ligand4.py script from MGLTools, ensuring standardized atom types and docking compatibility.

**Figure 1.**
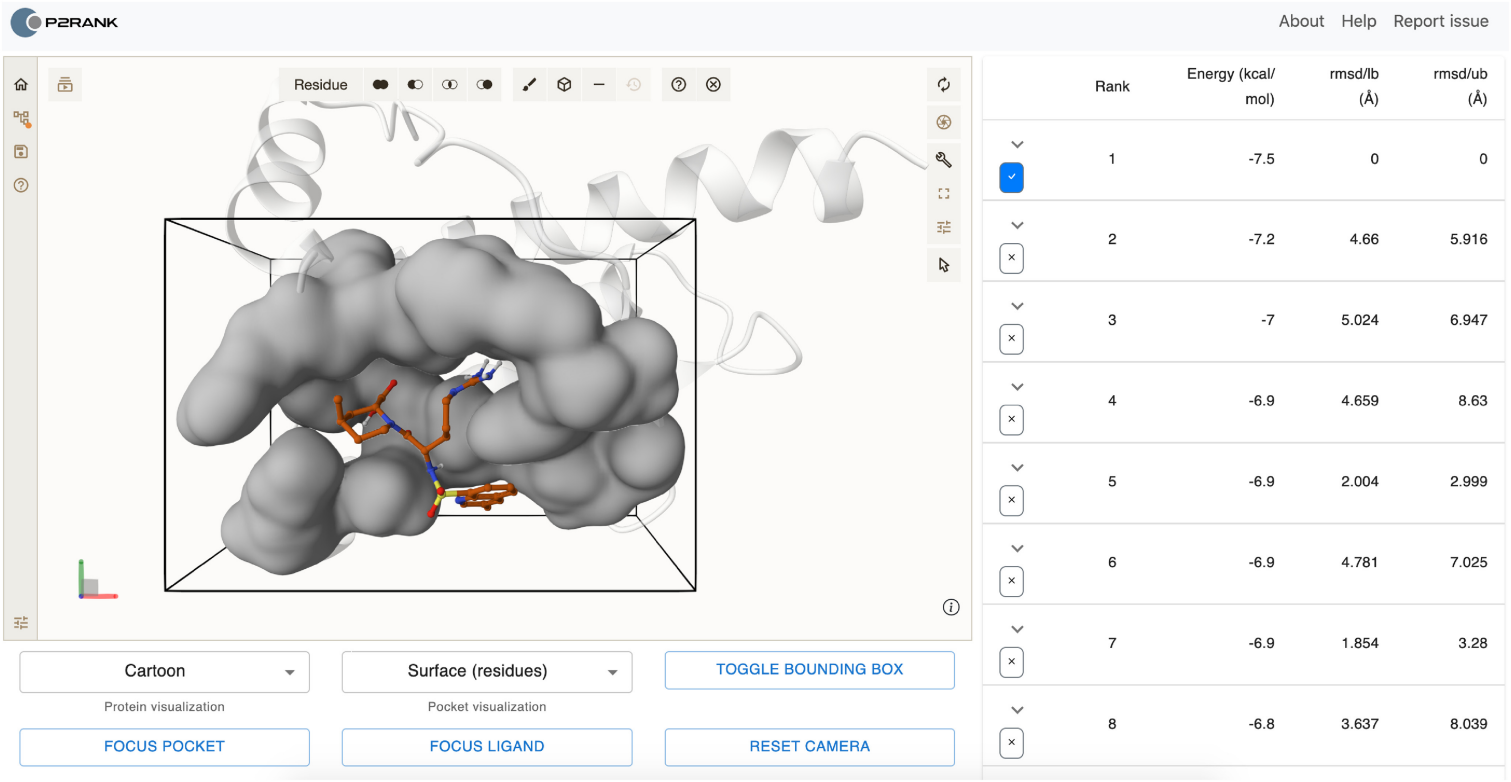
The graphical user interface for visualizing docking results. The image shows argatroban (PubChem CID: 10962209) bound to the human prion protein variant M166V (PDB ID: 1E1J). The displayed pose represents the top-ranked docking result.

The molecular docking visualization module, showcased in Fig. 1, provides an intuitive environment for visualizing the docking results. It integrates multiple interactive features that allow users to navigate molecular docking outputs efficiently. Users can browse and visualize multiple ligand binding poses identified in the docking process, along with their corresponding docking scores and RMSD (root mean squared deviation) values (relative to the best-scoring docking pose).

The graphical user interface supports various 3D structure representations, including cartoon, surface, and ball-and-stick models. Users can customize the visualization style for both the protein and the binding pocket. Ligands present in the original structure are shown in blue, while docked ligands are displayed in orange. A slider allows users to control the structure transparency. Quick navigation tools, such as “Focus Ligand,” “Focus Pocket,” and “Show/Hide bounding box,” help in structural alignment and visual inspection. Docking results are available for download in PDBQT format, ensuring compatibility with PyMOL and other molecular modeling tools.

Figure 1 demonstrates the docking results for argatroban (PubChem CID: 10962209) docked to the human prion protein variant M166V (PDB ID: 1E1J [27]). The figure shows the top-ranked docking pose, highlighted in orange, within the predicted binding pocket, displayed as a surface representation to provide a clear view of the binding site. PrankWeb4 yielded a binding energy of −8.072 kcal/mol for the top pose under the condition with evolutionary conservation enabled and the exhaustiveness parameter set to 32.

To contextualize this result, we performed a comparative docking analysis across three representative protein–ligand systems (1E1J + argatroban, 4HFP [28] + argatroban, and 1IEP [29, 30] + STI [30]) using other three online different AutoDock Vina-based platforms: SwissDock [18], Webina [31], and CBDock [19, 32]. Despite differences in input requirements, receptor/ligand preprocessing, and docking configuration, the predicted binding energies across platforms were largely consistent, typically varying within a 1–2 kcal/mol range (see “tool-to-tool range” rows in Supplementary Table S1). Detailed comparisons, including grid setup, binding energy distributions, and pocket coordinates, are provided in Supplementary Tables S1–S3.

## P2Rank improvements

The new version of PrankWeb also comes with P2Rank version 2.5, which is approximately twice as fast as the previous version. This improvement applies to both single-file processing, mainly due to faster model loading, and batch processing of large datasets, thanks to more efficient model inference and protein surface computation. The speedup was achieved by optimizing the numerical algorithm responsible for generating points on the solvent-accessible surface from the CDK library [33] and a complete reimplementation of the employed random forest algorithm.

Furthermore, P2Rank now generates an offline visualization script for UCSF ChimeraX [16], complementing the existing PyMOL visualization capabilities. The visualizations can be downloaded directly from PrankWeb for each prediction.

## Other improvements

**Figure 2.**
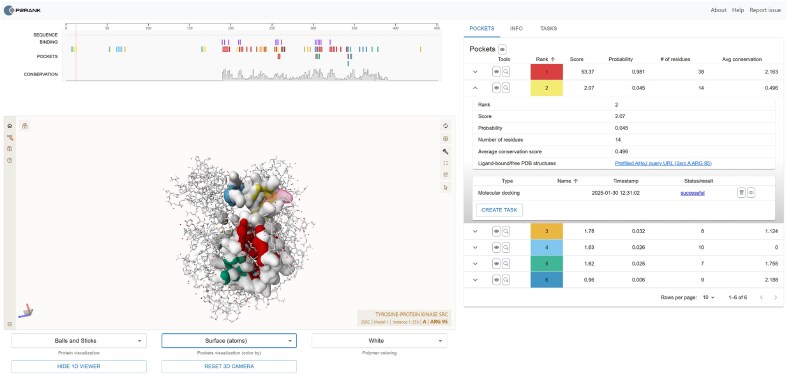
PrankWeb results visualization page. The view shows predicted binding sites on the human tyrosine kinase C-SRC (PDB ID 2SRC), available at https://prankweb.cz/viewer?id=2src&database=v3-conservation-hmm. Pockets are displayed using surface visualization, while the rest of the structure is shown as ball-and-stick. Different putative pockets are distinguished by color. In PrankWeb 4, we restructured the pocket information view so that it now shows precomputed statistics of the selected pocket, together with the results of the tasks, such as docking. The user can also directly start a task on a selected pocket.

PrankWeb originally used LiteMol [34] for 3D visualization; however, to enhance performance and improve the visual representation of structures, PrankWeb 4 transitioned to Mol* [35]. For sequence visualization, PrankWeb 4 utilizes the RCSB Saguaro 1D Feature Viewer [36]. All components of the website support interactive, synchronized visualization, allowing interaction between the 1D and 3D viewers in both directions, as shown in Fig. 2.

We have added an option in the structure information tab to display the P2Rank version used for the prediction, informing users about the specific version applied. Additionally, the exported ZIP file now includes the prediction in JSON format.

With the release of P2Rank 2.5, we have introduced a new database featuring precomputed PDB entries, enabling faster queries.

## Conclusion

Here, we introduced PrankWeb 4, a new version of the PrankWeb featuring a modular architecture, enabling pocket postprocessing modules that extend its functionality beyond binding site prediction. This modular architecture was used to develop a docking module, allowing users to explore ligand interactions directly within the platform.

The redesigned interface improves user interaction, enhances visualization, and incorporates a faster P2Rank backend with improved prediction versioning and expanded data export options.

These advancements make PrankWeb a more versatile tool for structural biology and drug discovery. In the future, we plan to focus on expanding modules and refining the user experience.

## Supplementary Material

gkaf421_Supplemental_Files

## Data Availability

The PrankWeb web server is publicly available at https://prankweb.cz/. The source codes are available at https://github.com/cusbg/p2rank-framework, https://doi.org/10.5281/zenodo.15319389, and https://doi.org/10.5281/zenodo.15303370.
