# Supplementary material for: PrankWeb 4: a modular web server for protein–ligand binding site prediction and downstream analysis
Source: Nucleic Acids Res. 2025 May 19;53(W1):W466–71. doi: 10.1093/nar/gkaf421 (PMC12230715; doi:10.1093/nar/gkaf421)
Supplement: gkaf421_Supplemental_Files [file gkaf421_supplemental_files.zip › Paper_PrankWeb4_SI_rev_KR.pdf]

SUPPORTING INFORMATION

PrankWeb4: A Modular Web Server for Protein-Ligand Binding Site Prediction and Downstream Analysis

Lukáš Polák[1], Petr Škoda[1], Kamila Riedlová[1], Radoslav Krivák[1,3], Marian Novotný[2] and David Hoksza[1*]

[1]Department of Software Engineering, Faculty of Mathematics and Physics, Charles University, Czech Republic

[2]Department of Cell Biology, Faculty of Science, Charles University, Czech Republic

[3]Institute of Organic Chemistry and Biochemistry of the Czech Academy of Sciences, Prague, Czech Republic

[*]corresponding author: david.hoksza@matfyz.cuni.cz

INTRODUCTION

This document provides supplementary data supporting the docking analyses presented in the main text. It includes comparative results obtained across three widely used online platforms (SwissDock [1], Webina [2], and CBDock [5, 6]) for three representative protein–ligand systems (1E1J + Argatroban, 4HFP + Argatroban, and 1IEP + STI [7]), alongside our results generated with PrankWeb4.

The supplementary tables (Tables S1–S3) summarize key metrics such as predicted binding energies, energy ranges, pocket coordinates, and box definitions. The comparisons were performed using consistent parameters (box center, box size, and exhaustiveness), with PrankWeb4 results evaluated under two conditions (with and without conservation) to assess its impact on docking performance. In the case of CBDock (a blind docking approach), it was not possible to define custom pocket coordinates or adjust the exhaustiveness parameter due to the platform's design and automation level. Nevertheless, CBDock was included to provide a contrasting perspective and to demonstrate performance under different docking philosophies. Where possible, results include multiple independent replicates and varying exhaustiveness settings. A complete set of individual docking energy values is provided at the end of this file. These materials offer a transparent basis for cross-platform validation in the context of these demonstrative case studies.

DOCKING STRATEGY AND INPUT PARAMETERS ACROSS PLATFORMS

To ensure consistency, pocket coordinates and box sizes for SwissDock and Webina were extracted from PrankWeb4's Pocket 1 predictions, under both conservation and non-conservation conditions. This approach enabled controlled comparison across platforms, as SwissDock and Webina require manual input of the docking region, whereas PrankWeb4 allows direct ligand docking into automatically predicted pockets. PrankWeb4 further supports SMILES input and offers pocket-switching via a user-friendly interface. In contrast, CBDock and CBDock2 operate as blind docking servers, automatically identifying cavities based on surface accessibility. They do not allow manual adjustment of the docking box or exhaustiveness parameters.

Each platform was used with its preferred input formats and default computational settings, unless otherwise specified. For SwissDock, the ligand was uploaded as a SMILES string, while the receptor structure was retrieved directly via PDB ID. Docking was carried out using the “Docking with AutoDock Vina” option. Webina accepted the receptor in .pdb format

and the ligand as a SMILES string; the correct pose option was not used. The docking was performed using 2 CPUs and no advanced parameters were applied beyond the default configuration. For CBDock2, the ligand was converted from SMILES to .sdf using Open Babel, and the receptor was uploaded in .pdb format. The number of cavities was set to 5, and other parameters were left as default. The original CBDock server was used analogously, with the receptor in .pdb format and the ligand in .sdf; five cavities were also requested.

Although all platforms rely on AutoDock Vina [3, 4] as the core docking engine, they differ in the specific version used, which may contribute to minor discrepancies in scoring behavior:

- PrankWeb4: AutoDock Vina v1.2.5
- SwissDock: AutoDock Vina v1.2.0
- Webina: AutoDock Vina v1.2.3
- CBDock: AutoDock Vina v1.1.2

These version differences, along with variations in input preprocessing and internal workflows, likely contribute to the minor variability observed in predicted results. Nevertheless, the binding energy values across tools were generally consistent, typically within a 1–2 kcal/mol window (see the 'tool-to-tool range' rows in Table S1). This consistency supports the robustness of PrankWeb4 when compared with other commonly used online tools.

STATISTICAL OVERVIEW OF BINDING ENERGY VARIABILITY

Table S1 provides an overview of binding energy predictions for Pocket 1 under two conditions (with and without evolutionary conservation), evaluated across the three tested protein–ligand systems. For each combination of tool and system, we report the observed energy range across different exhaustiveness values (2, 4, 8, and 32) and the average binding energy with standard deviation. Where possible, these values were derived from up to three independent replicates.

INTERPRETATION OF TOOL-TO-TOOL DIFFERENCES

The "tool-to-tool range" values (last row in each block) summarize the spread of average binding energies obtained from different platforms under identical conditions. These values typically remain within 1–1.5 kcal/mol, supporting cross-platform consistency in identifying top-

scoring binding sites. Notable deviations, such as the broader spread in the 1IEP + STI system without conservation, reflect differences in pocket recognition and ligand placement under more flexible or blind docking conditions.

Table S1. **Summary of binding energy values obtained for Pocket 1 using three online docking platforms (PrankWeb4, SwissDock, and Webina) across three protein-ligand systems.** For each system, results are shown under two conditions (with and without conservation). Pocket 1 coordinates and box sizes were extracted from PrankWeb4 predictions.

Protein + Ligand	Condition	Tool	Range[1] [kcal/mol]	Difference[2] [kcal/mol]	Average ± SD[3] [kcal/mol]
1E1J + Argatroban	Pocket 1 with conservation	PRANKWEB	-8.099 to -8.036	0.063	-8.070 ± 0.026
		SwissDock	-8.246 to -7.044	1.202	-7.979 ± 0.369
		Webina	-7.302 to -6.188	1.114	-6.724 ± 0.486
		Tool-to-tool range[4]			**1.345**
	Pocket 1 without conservation	PRANKWEB	-8.085 to -6.954	1.131	-7.759 ± 0.539
		SwissDock	-8.220 to -6.005	2.215	-7.461 ± 0.915
		Webina	-7.904 to -6.228	1.676	-7.000 ± 0.566
		Tool-to-tool range			**0.759**
4HFP + Argatroban	Pocket 1 with conservation	PRANKWEB	-8.559 to -8.479	0.080	-8.523 ± 0.035
		SwissDock	-9.520 to -9.351	0.169	-9.444 ± 0.048
		Webina	-10.668 to -8.356	2.312	-9.647 ± 0.701
		Tool-to-tool range			**1.123**
	Pocket 1 without conservation	PRANKWEB	-9.114 to -8.518	0.596	-8.680 ± 0.290
		SwissDock	-9.434 to -9.193	0.241	-9.344 ± 0.105
		Webina	-10.459 to -7.942	2.517	-9.152 ± 0.826
		Tool-to-tool range			**0.664**
1IEP + STI	Pocket 1 with conservation	PRANKWEB	-12.548 to -9.381	3.167	-11.368 ± 1.492
		SwissDock	-12.705 to -9.460	3.245	-11.092 ± 1.301
		Webina	-10.595 to -9.129	1.466	-10.013 ± 0.437
		Tool-to-tool range			**1.355**
	Pocket 1 without conservation	PRANKWEB	-9.829 to -8.770	1.059	-9.429 ± 0.460
		SwissDock	-9.662 to -9.293	0.369	-9.465 ± 0.159
		Webina	-13.322 to -7.724	5.598	-10.670 ± 1.434
		Tool-to-tool range			**1.241**

[1]Observed binding energy range (minimum to maximum) across different exhaustiveness settings. [2]The difference between maximum and minimum binding energy values. [3]Mean binding energy ± standard deviation (SD) calculated from multiple exhaustiveness settings (2, 4, 8 and 32) and, where possible,

calculated on three independent replicates. [4]Tool-to-tool range (across averages) refers to the difference between the highest and lowest average binding energy values among the three docking tools for each protein-ligand system and docking condition.

Together, these data demonstrate that despite slight variations, all evaluated tools converge on comparable docking results, and that PrankWeb4 provides competitive performance in terms of both energy prediction and reproducibility. The use of evolutionary conservation further contributes to more favorable and stable results, particularly visible in the 1IEP + STI case, where the predicted energy improved from –9.62 to –12.55 kcal/mol when conservation was enabled (see Table S2 for details).

COMPARISON WITH AUTODOCK VINA OFFICIAL TUTORIAL (1IEP + STI SYSTEM)

Table S2 provides a focused comparison of the best binding energy predictions for the 1IEP + STI system, calculated using different docking tools under various grid and exhaustiveness configurations. The top row lists the official AutoDock Vina tutorial [7] result (–14.72 kcal/mol), which serves as a reference. To replicate this setup, we used the same grid center and box size (20 × 20 × 20 Å) in Webina and SwissDock (where allowed), resulting in slightly less favorable energies (e.g., –12.57 kcal/mol and –10.40 kcal/mol, respectively), likely due to minor differences in ligand/receptor preparation or tool-specific docking behavior.

PrankWeb4 was used in two modes: with and without evolutionary conservation. Both modes predicted binding poses in different pocket locations, with grid sizes derived from the respective predicted Pocket 1 regions. In the conservation-based case, PrankWeb4 predicted a binding energy of –12.548 kcal/mol, which closely matches the tutorial benchmark. The non-conservation setup yielded a weaker predicted binding (–9.615 kcal/mol), indicating that the conserved pocket provides a more favorable and specific binding environment.

SwissDock was not able to compute results for the larger PrankWeb4-derived boxes due to time restrictions on the server. Webina, however, accepted all configurations and allowed direct comparison across all tested boxes. In all cases, differences in energy values reflect variations in pocket location, grid size, and ligand/receptor preprocessing. We also tested .pdbqt input files for both the ligand and receptor, prepared using our standard protocol in Webina. However, no significant differences in predicted binding energies were observed compared to the default input formats, i.e., SMILES for ligands and receptor structures obtained directly from the PDB database or automatically retrieved via PDB ID (in case SwissDock).

Despite these differences, the relative ranking of predicted binding affinities remains consistent, particularly when comparing conserved versus unconserved conditions. This further supports the practical applicability of PrankWeb4's pocket prediction strategy in virtual screening workflows.

Table S2. **Best binding energy value for the 1IEP + STI system obtained using PrankWeb4, Webina, SwissDock, and the official AutoDock Vina tutorial settings**[1]**.** For each docking run, the box center, box size, exhaustiveness, and predicted binding energy are shown. Input parameters were either derived from the AutoDock Vina tutorial or from Pocket 1 predicted by PrankWeb4, under both conservation and non-conservation conditions.

**1IEP + STI ligand – comparison with AutoDock Vina official tutorial**				
**Run / Tool**	**Box center (x, y, z) [Å]**	**Box size [Å]**	**Exhaustiveness**	**Best binding energy (kcal/mol)**
AutoDock Vina tutorial[1]	(15.19, 53.903, 16.917)	20 x 20 x 20	32	-14.72
SwissDock	(15.19, 53.903, 16.917)	20 x 20 x 20	32	-12.57
Webina	(15.19, 53.903, 16.917)	20 x 20 x 20	32	-10.401
PrankWeb4 (Pocket 1, with conservation)	(16.9132, 54.5702, 16.1823)	25 x 25 x 25	32	-12.548
SwissDock	(16.9132, 54.5702, 16.1823)	25 x 25 x 25	not allowed[2]	-
Webina	(16.9132, 54.5702, 16.1823)	25 x 25 x 25	32	-10.215
PrankWeb4 (Pocket 1, without conservation)	(14.0459, 99.7595, 53.7838)	18 x 18 x 18	32	-9.615
SwissDock	(14.0459, 99.7595, 53.7838)	18 x 18 x 18	not allowed[2]	-
Webina	(14.0459, 99.7595, 53.7838)	18 x 18 x 18	32	-11.824

[1]Source: AutoDock Vina tutorial webpage. [2]Online SwissDock calculations for these systems were not allowed by the "Check parameters" available on the webpage due to the SwissDock computational time restriction required for these calculations.

COMPARISON OF POCKET DEFINITIONS AND ENERGIES ACROSS TOOLS.

Table S3 compares the top-ranked predicted binding sites (Pocket 1 or Cavity 1) across three systems (1E1J + Argatroban, 4HFP + Argatroban, and 1IEP + STI), as identified by PrankWeb4 and CBDock. Although the binding sites were identified using distinct methodologies, their predicted pocket centers often localize near similar structural regions – particularly in the 1E1J and 4HFP systems, where coordinate differences are sometimes within only a few angstroms. However, binding energies and box sizes differ more substantially. CBDock tends to assign larger, more general boxes (typically 23–30 Å in this case). In contrast, PrankWeb4 adapts the box size to the predicted pocket volume, often resulting in smaller, more focused docking regions.

PrankWeb4 predictions with evolutionary conservation consistently yielded more favorable binding energies than those without conservation, supporting the idea that conserved regions provide more functionally relevant binding sites. For example, in the 1IEP + STI system, the energy difference between conservation and non-conservation modes is nearly 3 kcal/mol (-12.548 kcal/mol vs. -9.615 kcal/mol). Despite methodological differences, this suggests that both evolutionary-guided and blind docking approaches can yield comparable predictions when applied thoughtfully. The choice of tool may thus depend on the desired balance between automation and biological relevance.

Table S3. **Comparison of Pocket 1 binding energies and coordinates across three different systems: PrankWeb4 (with/without conservation) vs. CBDock.**

Protein + Ligand	Pocket[1]	Tool	Binding energy [kcal/mol]	Pocket 1 coordinates (x, y, z) [Å]	Approx. Size [Å]
1E1J + Argatroban	Pocket 1	PrankWeb4 (with conservation, exhaustiveness 32)	-8.072	(-3.8736, -8.2774, 5.1638)	20 x 20 x 20
		CBDock2	-7.4 to -7.1	(-6, -7, 5)	23 x 23 x 23
		CBDock	-7.4 to -7.0	(-6, -7, 5)	23 x 23 x 23
		PrankWeb4 (without conservation, exhaustiveness 32)	-8.038	(-6.5481, -7.3498, 3.9936)	15 x 15 x 15
4HFP + Argatroban	Pocket 1	PrankWeb4 (with conservation, exhaustiveness 32)	-8.559	(8.9727, 15.8043, -1.3537)	19 x 19 x 19
		CBDock2	-9.4 to -9.2	(55, -30, -8)	23 x 23 x 23
		CBDock	-9.8 to -9.5	(55, -30, -8)	23 x 23 x 23
		PrankWeb4 (without conservation, exhaustiveness 32)	-8.535	(8.2270, 17.9273, -1.1270)	21 x 21 x 21
1IEP + STI	Pocket 1	PrankWeb4 (with conservation, exhaustiveness 32)	-12.548	(16.9132, 54.5702, 16.1823)	25 x 25 x 25
		CBDock2	-11.9 to -9.7	(13, 56, 15)	30 x 30 x 30
		CBDock	-13.3 to -11.4	(13, 56, 15)	30 x 30 x 30
		PrankWeb4 (without conservation, exhaustiveness 32)	-9.615	(14.0459, 99.7595, 53.7838)	18 x 18 x 18

[1]The top five pockets (PrankWeb4) or cavities (CBDock) identified by each tool, along with their corresponding binding energies, are available in the Supplementary Data file, which includes most parameters and results used in the analysis.

CONCLUSION

This Supporting Information provides comparative analyses of binding energy predictions, docking parameters, and pocket characteristics across several online platforms and protein–ligand systems. The results demonstrate that PrankWeb4 performs well in terms of both accuracy and usability, with evolutionary conservation contributing positively to pocket quality and binding energy values. Full docking results and parameters are provided in the accompanying dataset for transparency.

REFERENCES

[1] Bugnon, M., Röhrig, U. F., Goullieux, M., Perez, M. A., Daina, A., Michielin, O., & Zoete, V. (2024). SwissDock 2024: major enhancements for small-molecule docking with Attracting Cavities and AutoDock Vina. *Nucleic acids research*, *52*(W1), W324-W332.

[2] Kochnev, Y., Hellemann, E., Cassidy, K. C., & Durrant, J. D. (2020). Webina: an open-source library and web app that runs AutoDock Vina entirely in the web browser. *Bioinformatics*, *36*(16), 4513-4515.

[3] Trott, O., & Olson, A. J. (2010). AutoDock Vina: improving the speed and accuracy of docking with a new scoring function, efficient optimization, and multithreading. *Journal of computational chemistry*, *31*(2), 455-461.

[4] Eberhardt, J., Santos-Martins, D., Tillack, A. F., & Forli, S. (2021). AutoDock Vina 1.2. 0: New docking methods, expanded force field, and python bindings. *Journal of chemical information and modeling*, *61*(8), 3891-389.

[5] Liu, Y., Grimm, M., Dai, W. T., Hou, M. C., Xiao, Z. X., & Cao, Y. (2020). CB-Dock: a web server for cavity detection-guided protein–ligand blind docking. *Acta Pharmacologica Sinica*, *41*(1), 138-144.

[6] Liu, Y., Yang, X., Gan, J., Chen, S., Xiao, Z. X., & Cao, Y. (2022). CB-Dock2: improved protein–ligand blind docking by integrating cavity detection, docking and homologous template fitting. *Nucleic acids research*, *50*(W1), W159-W164.

[7] Forli, S., Huey, R., Pique, M. E., Sanner, M. F., Goodsell, D. S., & Olson, A. J. (2016). Computational protein–ligand docking and virtual drug screening with the AutoDock suite. Nature protocols, 11(5), 905-919.
